# Transcriptome Analysis of Pacific White Shrimp (*Litopenaeus vannamei*) Hepatopancreas in Response to Taura Syndrome Virus (TSV) Experimental Infection

**DOI:** 10.1371/journal.pone.0057515

**Published:** 2013-02-28

**Authors:** Digang Zeng, Xiuli Chen, Daxiang Xie, Yongzhen Zhao, Chunling Yang, Yongmei Li, Ning Ma, Min Peng, Qiong Yang, Zhenping Liao, Hui Wang, Xiaohan Chen

**Affiliations:** Guangxi Key Laboratory of Aquatic Genetic Breeding and Healthy Aquaculture, Guangxi Institute of Fisheries, Nanning, China; Institut Pasteur, France

## Abstract

**Background:**

The Pacific white shrimp, *Litopenaeus vannamei*, is a worldwide cultured crustacean species with important commercial value. Over the last two decades, Taura syndrome virus (TSV) has seriously threatened the shrimp aquaculture industry in the Western Hemisphere. To better understand the interaction between shrimp immune and TSV, we performed a transcriptome analysis in the hepatopancreas of *L. vannamei* challenged with TSV, using the 454 pyrosequencing (Roche) technology.

**Methodology/Principal Findings:**

We obtained 126919 and 102181 high-quality reads from TSV-infected and non-infected (control) *L. vannamei* cDNA libraries, respectively. The overall *de novo* assembly of cDNA sequence data generated 15004 unigenes, with an average length of 507 bp. Based on BLASTX search (E-value <10−5) against NR, Swissprot, GO, COG and KEGG databases, 10425 unigenes (69.50% of all unigenes) were annotated with gene descriptions, gene ontology terms, or metabolic pathways. In addition, we identified 770 microsatellites and designed 497 sets of primers. Comparative genomic analysis revealed that 1311 genes differentially expressed in the infected shrimp compared to the controls, including 559 up- and 752 down- regulated genes. Among the differentially expressed genes, several are involved in various animal immune functions, such as antiviral, antimicrobial, proteases, protease inhibitors, signal transduction, transcriptional control, cell death and cell adhesion.

**Conclusions/Significance:**

This study provides valuable information on shrimp gene activities against TSV infection. Results can contribute to the in-depth study of candidate genes in shrimp immunity, and improves our current understanding of this host-virus interaction. In addition, the large amount of transcripts reported in this study provide a rich source for identification of novel genes in shrimp.

## Introduction

Taura syndrome virus (TSV) is a contagious viral disease of penaeid shrimp [1]. Over the last two decades, TSV has seriously threatened the shrimp aquaculture industry and caused serious economic losses [2], [3]. In cultured Pacific white shrimp (*Litopenaeus vannamei*), which has become the major aquacultured crustacean species in the Western Hemisphere [4], TSV can cause a cumulative mortality ranged from 40 to >90% [5]. The survival shrimp of TSV infections may carry the virus for life [6], [7]. TSV was first discovered in South America, but has later spread to North America, Hawaii and Asia [8]–[11]. TSV is a small, simple RNA virus. The genome of TSV is a positive-sense singlestranded RNA of 10.2 kb containing two open reading frames [12]. Although considerable progress has been made in the molecular characterization of TSV [13], no effective cure for this disease has yet been found [14]. Shrimp lack an acquired immune system. Their defense is considered to depend entirely on an innate, non-adaptive mechanism to defense invasion by pathogens [15]. An understanding of the host-pathogen interaction will be helpful in controlling infectious diseases in shrimp[15]. Therefore, the molecular response of shrimp to viral infection is becoming an increasingly important subject for study [16]. Recently, the molecular mechanism of the shrimp-virus interaction has made notable progress [17], many genes involved in viral infection in shrimp were found, such as lectins, antimicrobial peptide, blue blood protein and superoxide dismutase [18]–[21]. Most of these genes were identificated using the suppression subtractive hybridization (SSH) technology. SSH is an effective approach for identifying differentially expressed genes among different biological processes[22]. However, subtractive hybridization does not provide a quantitative measure of expression differences, and its experimental results often contain a large number of false positives [23]. Recently, the high-throughput sequencing technologies, such as the Illumina Genome Analyzer, the Applied Biosystems Solid platform, and the 454 Life Sciences (Roche) pyrosequencing platform, provide a rapid and high-throughput method to identify differentially expressed genes and their expression profile [24]–[26].

Identification of host genetic factors in response to pathogen is of great significance for shrimp breeding and production. However, information on the host genes involved in TSV pathogenesis is still limited. To our knowledge, there is no previous report of isolating genes that are involved in TSV infection. In this study, we performed a transcriptome analysis of the hepatopancreas of *L. vannamei* challenged with TSV, using a high-throughput sequencing method (Roche 454 pyrosequencing). The aim of this study was to discover new genes involved in TSV infection, and better understand the virus-host interaction. Furthermore, the high-throughput sequencing will produce a large number of transcripts in this study, providing a strong basis for future genomic research on shrimp.

## Materials and Methods

### Shrimp, Virus and Challenge

*L. vannamei* from a specific pathogen-free (SPF) line (High Health Aquaculture, Kona, Hawaii, USA) were used in this study. The shrimp (11–12 g body weight) were provided from the National and Guangxi Vannamei Genetic Breeding Center, Guangxi Province, China, and held in the environmentally controlled 1000-liter glass saltwater tanks (32-ppt salinity, 25 to 26°C) and fed an artificial pellet feed. The shrimp were randomly sampled and tested by PCR for certify to be free of TSV by PCR[5]. In the challenge experiment, there were two shrimp groups: 1 TSV challenge group, and 1 negative control group (20 shrimp each group). The challenge group was fed once a day for 3 consecutive days with minced virus infected tail tissue at 10% of their body weight. In parallel, the negative control group was fed once a day throughout the test period of 3 days with minced PCR-confirmed [5] healthy tail tissue at 10% of their body weight. At 72 hours after infection, the hepatopancreas tissues of shrimp were collected in cryotubes and stored in liquid nitrogen for later RNA extraction.

### RNA Extraction, cDNA Library Construction, and Deep Sequencing

Total RNAs were extracted from TSV-infected and non-infected shrimp hepatopancreas using TriReagent (Qiagen), and the mRNAs were purified from the total RNAs using the PolyATtract mRNA isolation systems (Promega) following the manufacturer’s instructions. Integrity and size distribution were checked with Bioanalyzer 2100 (Agilent technologies, USA). Equal amounts of the high-quality mRNA samples from each group were then pooled for cDNA synthesis and sequencing. The normalized cDNA library was prepared following the 454 mRNA pyrosequencing sample preparation procedure (Roche, IN, USA). Library construction and pyrosequencing was carried out by Beijing Autolab Biotechnology Co., Ltd. on a 454 GS FLX system (Roche).

### *De novo* Assembly and Functional Annotation

Raw sequencing reads were quality trimmed, and adaptor sequences were removed before the assembly. After removal of low quality reads, processed reads were assembled using CAP3 software with default parameters[27]. The overall assembly was performed using the combined sequence data for both the TSV-infected sample and the non-infected sample. The contigs and singletons were generally referred to as unigenes. Subsequently, the unigenes were subjected to BLASTX similarity search against NCBI non-redundant protein database and the swissprot database using BLASTALL programs with an E-value threshold of 10^−5^ [28]. All annotated unigenes were used to determine the COG term, GO term and KEGG pathway with a cut-off E-value of 10^−5^ using BLASTX[29], [30].

### Identification of Differentially Expressed Genes

For differential gene expression analyzes, RPKM (reads per kilobase per million reads) were used as the value of normalized gene expression levels[31]. Statistical comparison of RPKM values between the TSV-infected sample and the non-infected sample was conducted using a web tool IDEG6 (http://telethon.bio.unipd.it/bioinfo/IDEG6_form/) [32]. FDR (false discovery rate) <0.001 was used as the threshold of P-value in multiple test to judge the significance of gene expression difference [33]. Genes were considered differentially expressed in a given library when the p-value ≤0.001 and a greater than two-fold change (absolute value of log2 ratio >1) in expression across libraries was observed.

### Quantitative RT-PCR Analysis

To validate our 454 sequencing data, six differentially expressed *L. vannamei* genes (cathepsin-L, arginine kinase, fatty acids binding protein, alternative splicing factor, sorbitol dehydrogenase and hemocyanin) were selected for quantitative RT-PCR analysis, using the same RNA samples as for the transcriptome profiling. Primers were designed using the Primer5 software (Premier Biosoft International) (Table S1). First strand cDNA was synthesized from 1 μg of RNA using M-MuLV reverse transcriptase (Qiagen). The qPCR reaction mixture (20 μL) consisted of 2× Power SYBR Green PCR Master mix, 0.9M each of the forward and reverse primers, and 1 μL of template cDNA. PCR amplification was performed under the following conditions: 50°C for 2 min and 95°C for 30 s, followed by 40 cycles of 95°C for 15 s and 62°C for 1 min, and a final extension at 72°C for 5 min.

### Identification of Microsatellites

All the assembled cDNA contigs from both the infected library and the control library were used for identification of microsatellites. All types of microsatellites from dinucleotides to hexanucleotides were detected using the MISA software [34] with default parameters (for all repeat types, minimum total length = 15 bp and minimum repeats = 3). Primers were designed using the primer3 software [35].

## Results

### Pyrosequencing and Assembly

To identify the genes involved in *L. vannamei* response to TSV infection, we created two cDNA libraries from pooled mRNAs extracted from the hepatopancreas of TSV-infected and non-infected (control) groups, respectively. The two libraries were subjected to a pyrosequencing run on the 454 GS FLX system, resulting in 131745 (TSV-infected sample) and 110721 (non-infected sample) raw reads, respectively. Files containing these data were deposited in the Short Read Archive of the National Center for Biotechnology Information (NCBI) with accession numbers of SRR554365 (TSV-infected) and SRR556131 (non-infected). After filtering for adaptors and low-quality sequences, the TSV-infected library generated 126919 cleaned reads, ranging from 41 bp to 620 bp, with the average length of 367 bp and N50 length of 454 bp (Table S2). In the non-infected library, a total of 102181 cleaned reads were obtained, ranging from 45 bp to 619 bp, with the average length of 364 bp and N50 length of 454 bp (Table S3). The overall assembly was performed using the combined cleaned reads from the two libraries. *De novo* assembly using the CAP3 software produced 15004 unigenes (including contigs and singletons) with an average length of 507 bp, ranging from 42 to 8750 bp (Table 1).

**Table 1:** Length distribution of assembled unigenes.

Length (bp)	Number of sequences	Percentage
1–500	7232	48.20%
500–1000	6674	44.48%
1000–1500	736	4.91%
1500–2000	250	1.67%
≥2000	112	0.75%
Total	15004	
N50 length	539 bp	
Average length	507 bp	
Maximum length	8750 bp	

### Functional Annotation

All unigenes were compared with the Swiss-Prot and the NCBI non-redundant (NR) protein databases for functional annotation by using BLASTX with an e-value threshold of 10^−5^. Among the 15004 unigenes from both the TSV-infected library and the non-infected library, 4400 (29.33%) showed significant matches (E-value ≤10^−5^) in the Swiss-Prot database. An additional 10412 (69.39%) unigenes showed significant matches (E-value ≤10^−5^) in the NR database. In total, 10425 (69.50%) unigenes were annotated in Swiss-Prot or NR database.

**Figure 1:**
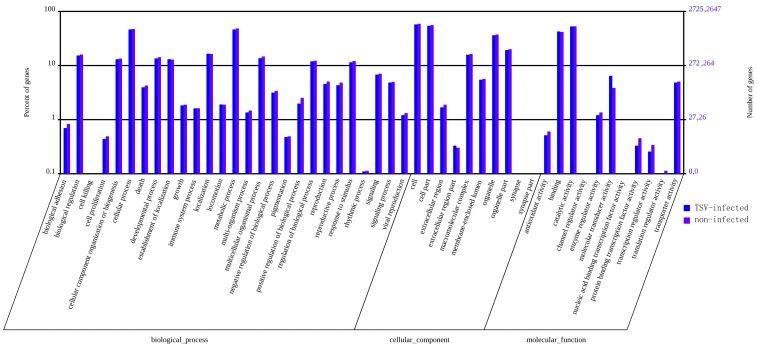
GO annotations of unigenes in the TSV-infected and non-infected *Litopenaeus vannamei* cDNA libraries. Most unigenes can be divided into three major categories, including biological process, cellular component, and molecular function.

Gene ontology (GO) analysis was performed with the unigenes from both the infected library and the control library. A total of 6567 and 6604 unigenes map to biological processes, 2977 and 2828 unigenes map to molecular functions, and 5206 and 5222 unigenes map to cellular components in the TSV-infected library and non-infected control library, respectively (Table S4). The functional distribution of the genes of the two libraries was similar. In both libraries, most of the corresponding biological process genes were involved in metabolic processes and cellular processes. Most of the molecular function genes were associated with catalytic activity, binding, and molecular transducer activity; most of the cellular component genes encode proteins associated with cell, parts of cell and cell organelles (Figure 1).

**Figure 2:**
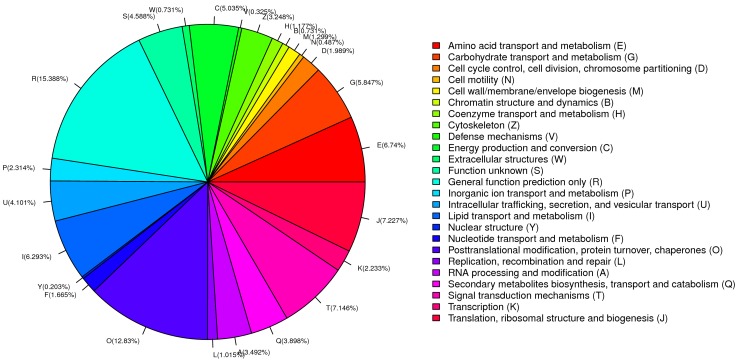
COG function classification of unigenes in the TSV-infected and non-infected *Litopenaeus vannamei* cDNA libraries. All putative proteins were aligned to the COG database and can be classified functionally into at least 25 molecular families.

We also searched the annotated sequences for the genes involved in COG classification. Among the 25 COG categories, the cluster for ‘General function prediction only’ (15.388%) represented the largest group, followed by the ‘Posttranslational modification, protein turnover, chaperones’ (12.83%) and ‘Translation, ribosomal structure and biogenesis’ (7.227%) clusters (Figure 2).

To identify the biological pathways that are active in *L. vannamei*, we mapped all the unigenes to the referential canonical pathways in the KEGG database. A total of 14496 unigenes were assigned to 174 KEGG pathways (Table S5). The top 10 pathways are metabolic pathways (1762 members), phagosome (1035 members), focal adhesion (815 members), tight junction (779 members), adherens junction (768 members), biosynthesis of secondary metabolites (624 members), lysosome (374 members), ribosome (320 members), oxidative phosphorylation (267 members), and tyrosine metabolism (217 members).

In summary, for functional annotation, all the unigenes were searched against NR, Swissprot, GO, COG, and KEGG databases by BLASTX with a cut-off E-value of 10^−5^. By this method, 10425 unigenes (69.50% of all unigenes) returned an above cut-off BLAST result (Table 2). Of them, 10412 unigenes were annotated by NR (69.39%), and 4400 (29.33%), 3397 (22.64%), 3979 (26.52%), and 3393 (22.61%) unigenes by SwissProt, GO, COG and KEGG, respectively.

**Table 2:** Annotation of unigenes.

Database	Number of annotated unigenes	Percentage of annotated unigenes
Nr	10412	69.39%
Swissprot	4400	29.33%
GO	3397	22.64%
COG	3979	26.52%
KEGG	3393	22.61%
Total	10425	69.50%

### Identification of Differentially Expressed Genes

To identify differentially expressed genes potentially involved in TSV infection, we constructed two normalized cDNA libraries from pooled mRNAs extracted from the hepatopancreas of TSV-infected and non-infected groups, respectively. Subsequently, these libraries were sequenced by 454 GS FLX technology. Comparison of gene expression revealed 1311 genes differentially expressed in TSV-infected shrimp compared to the control, including 559 up-regulated genes and 752 down-regulated genes. The number of down-regulated genes is much larger than that of up-regulated genes, which might be consistent with the observation that viral-infected individuals of shrimp are less active. Among the 1311 differentially expressed genes, 1061(80.93%) genes were well annotated, whereas the remaining 250(19.07%) genes had low sequence homology to known sequences in public databases, suggesting that they might be putative novel genes in *L. vanname* involved in the response to TSV infection. All differentially expressed unigenes with their Nr, Nt, Swissprot, GO, COG, KEGG and ORF analysis are presented in additional Table S6.

**Figure 3:**
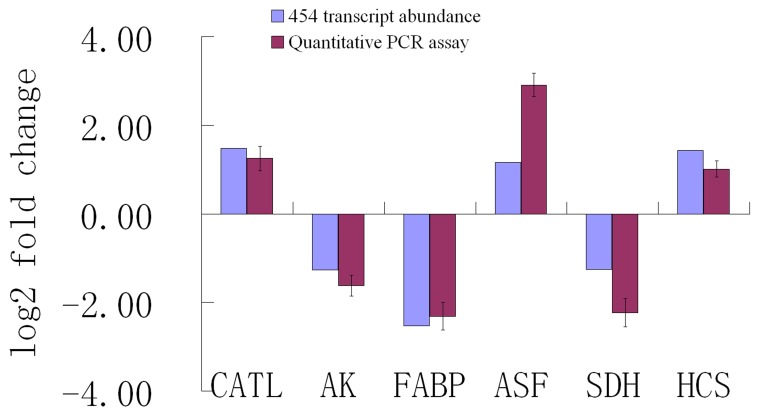
Comparison of the expression profiles of selected genes as determined by 454 sequencing (blue) and qRT-PCR (red). Target gene abbreviations are as follows: CATL - cathepsin L, AK - arginine kinase, FABP - fatty acids binding protein, ASF - alternative splicing factor, SDH - sorbitol dehydrogenase, HCS - hemocyanin.

To validate our RNA-seq results, six differentially regulated genes with the different total transcript reads (range 44–1076) were selected for quantitative real time-PCR (qRT-PCR) analysis. The results indicate that the qRT-PCR analysis of the relatively high abundant genes (>500 reads) agrees well with the 454 sequencing analysis. For example, based on 454 sequencing analysis, cathepsin-L (CATL), arginine kinase (AK) and fatty acids binding protein (FABP) were differentially regulated 1.48, −1.26 and −2.53 log2-fold, respectively, and showed 1.25, −1.62 and −2.31 log2-fold changes, respectively in qRT-PCR analyses (Figure 3). However, the qRT-PCR analysis of the relatively low abundant genes (<500 reads), including alternative splicing factor (ASF), sorbitol dehydrogenase (SDH) and hemocyanin (HCS), do not match the 454 sequencing analysis perfectly, even if it shows similar trends in up- or down-regulation of genes analysised by 454 sequencing (Figure 3). Nevertheless, qRT-PCR analysis confirmed the change direction detected by the 454 sequencing analysis.

### Candidate Genes Involved in *L. vanname* Immune Response

Among the genes that were found to be differentially expressed in the TSV-infected shrimp compared to non-infected controls, several are involved in various processes of animal immune response (Table 3). These were classified under 8 functions, including antiviral proteins, antimicrobial proteins, proteases, protease inhibitors, signal transducers and transcription factors, cell death and cell adhesion. Antiviral proteins are proteins that are induced by interferon in virus-infected human or animal cells and mediates interferon inhibition of virus replication [36]. Among the differentially expressed genes homologous to antiviral proteins, we found that a homolog of Zinc finger CCCH-type antiviral protein was significantly up regulated in TSV-infected shrimp compared to non-infected controls. The up-regulation of this gene after viral infection suggests that it may be involved in shrimp immune response. Antimicrobial proteins are an important component of the natural defenses of most living organisms against invading pathogens [15]. They interfere with microbial integrity or metabolism by targeting structures or nutrients specific to microbes [37]. Of the antimicrobial proteins identified in this study, Lysozyme and Histone H2A were significantly up regulated after TSV infection, indicating they may play important roles in shrimp defense against virus. Also of interest for the study of virus-host interactions is the identification of genes involved in signal transduction, as signal transduction molecules have been suggested to play important roles in viral recognition and replication [38]. We identified 8 differentially expressed genes involved in signal transduction, including P38 mapk, Map kinase-interacting serine/threonine, Serine/threonine-protein phosphatase alpha-1 isoform, Senescence-associated protein, Transmembrane BAX inhibitor motif-containing protein 4, C-type lectin, Innexin, and Fatty-acid amide hydrolase 2. All of these genes were up regulated. The other category of genes that are involved in transcriptional control, cell adhesion and cell death processes, may also play important roles during the TSV infection, as processes regulated by these genes have been suggested to modulate phagocytic events, cellular remodeling, recruitment of immune cells to sites of insult, and extracellular immune cascades such as the melanization response [39].

**Table 3:** Candidate genes involved in *L. vanname* immune response.

Category or gene ID	Homologous function	Species	FC*
**Antiviral**			
RL-all_c1285	Zinc finger CCCH-type antiviral protein	*Gallus gallus*	2.89
**Antimicrobial**			
RL-all_rep_c8569	Lysozyme	*Penaeus semisulcatus*	3.91
RL-all_rep_c13018	Histone H2A	*Nasonia vitripennis*	3.37
**Proteases**			
RL-all_c568	Clip domain serine proteinase	*Penaeus monodon*	5.05
RL-all_rep_c8737	Cathepsin L	*Penaeus monodon*	4.65
RL-all_c14	Cathepsin D	*Penaeus monodon*	2.75
RL-all_c8173	26S protease regulatory	*Nasonia vitripennis*	1.75
**Protease inhibitors**			
RL-all_rep_c13198	Insulin-like growth factor-binding protein 7 precursor	*Caligus rogercresseyi*	−1.57
RL-all_rep_c13202	Kazal-type serine proteinase inhibitor 4	*Procambarus clarkii*	−1.57
**Signal transduction**			
RL-all_c5388	P38 mapk	*Aedes aegypti*	5.42
RL-all_c92	Map kinase-interacting serine/threonine	*Scylla paramamosain*	1.62
RL-all_c948	Serine/threonine-protein phosphatase alpha-1 isoform	*Camponotus floridanus*	1.01
RL-all_rep_c12239	Senescence-associated protein	*Plasmodium yoelii*	2.33
RL-all_c7341	Transmembrane BAX inhibitor motif-containing protein 4	*Caligus rogercresseyi*	1.48
RL-all_rep_c12462	C-type lectin	*Penaeus monodon*	2.07
RL-all_c1259	Innexin	*Ixodes scapularis*	1.75
RL-all_c269	Fatty-acid amide hydrolase 2	*Camponotus floridanus*	1.92
**Transcriptional control**			
RL-all_c1160	RNA-binding protein 39	*Camponotus floridanus*	1.07
RL-all_c786	Glucose-regulated protein 78	*Fenneropenaeus chinensis*	−1.02
**Cell death**			
RL-all_rep_c8487	Apoptosis-inducing factor 1	*Branchiostoma floridae*	1.33
RL-all_c5051	Caspase	*Litopenaeus vannamei*	−1.57
RL-all_c8198	Beclin	*Camponotus floridanus*	−2.22
RL-all_c182	ATP binding cassette transmembrane transporter	*Litopenaeus vannamei*	−1.57
RL-all_c14	Lysosomal aspartic protease	*Penaeus monodon*	2.75
**Cell adhesion**			
RL-all_c438	Hemicentin-2	*Aedes aegypti*	1.75
RL-all_rep_c13362	Peritrophic membrane protein 1	*Holotrichia oblita*	−1.17

* fold changes (log2 ratio) in gene expression.

### Identification of Microsatellites

Microsatellites (or simple sequence repeats, SSRs) are repetitive sequence motifs of 1–6 bp [40], [41]. Although they are widely used as molecular markers due to their variability and abundance in the genome, codominant expression and inheritance in a Mendelian fashion [42], only a limited number of microsatellite sequences have been reported for *L.vannamei* [43]. In this study, we obtained 770 microsatellites, of which 23.90% were di-nucleotide repeats (184), followed by 36.88% tri-nucleotide repeats (284) and 36.23% tetra-nucleotide repeats (279), as well as 2.99% penta-nucleotide repeats (23) (Table S7). We also designed 497 primer sets using the Primer3 program (Table S8). These identified microsatellites have potential utility to genetic mapping, population structure and gene flow studies of *L. vannamei*.

## Discussion

Taura syndrome (TS) is a major cause of shrimp mortality in cultured *L. vannamei* in the Americas and Asia [1]. Although there are many published reports of characterization and detection of TSV, little is known about the interaction between this virus and shrimp. Understanding the interaction between host and its pathogen is useful, not only for studies on the molecular immune mechanisms, but also for agricultural practice that aims to provide a theoretical basis for developing effective strategies to prevent viral disease. Roche 454 RNA sequencing (RNA-Seq) is a powerful new method for discovering novel genes and investigating gene expression patterns, especially in non-model organisms that do not have sequenced genomes[44]. Like many other crustacean species with significant economic value, *L. vannamei* lacks a complete genome sequences and most other genetic tools and resources. In this study, we used the 454 RNA-Seq to investigate the gene expression changes associated with the TSV infection. We identified a total of 15004 unigenes in *L. vannamei*, 4579(30.52%) of which were new transcripts compared to known genes in public databases. Comparative analysis of transcriptome changes between TSV-infected and non-infected shrimp revealed 1311 differentially expressed genes, of which 559 genes were up-regulated and 752 down-regulated. Our sequencing data analyses indicate that TSV infection has a significant impact on the transcriptome profile of *L. vannamei* hepatopancreas.

Among the differentially expressed genes found in this study, several had been previously reported to be involved in the shrimp response against white spot syndrome virus (WSSV), such as C-type lectin and hemocyanin [15], [17], [45]–[48]. Animal C-type lectins play important roles in innate immunity to recognize and eliminate pathogens efficiently[49]. In invertebrates, C-type lectins are involved in non-self immune recognition and pathogen phagocytosis through opsonization[50]. Several studies reported the expression of C-type lectins in shrimp hepatopancreas was greatly affected after challenge by WSSV[20], [51]–[53]. In this study, we found 15 unigenes homologous to C-type lectins, and their expression exhibited to change significantly after TSV infection. Hemocyanin is another well-known immune-related gene previously reported to be involved in viral infection [54]. Hemocyanins are the oxygen-transporting proteins in arthropods and molluscs[55]. Hemocyanins have the defense-related functions that are mediated through phenoloxidase activity. Several previous studies reported that hemocyanins in shrimp were greatly over expressed during WSSV infection[56]–[58]. Similarly, several other previously reported differentially expressed genes, such as heat shock protein, lysozyme and fatty acid-binding protein, strongly up-regulated in shrimp challenged with WSSV[59]–[61]. In the present study, up-regulation of these genes have also been observed in shrimp challenged with TSV. It suggests that these genes might have the similar expression pattern in response to virus infection, regardless of the virus species.

Although some of the differentially expressed genes found in this study had not been previously reported to be involved in virus-host interaction, they were annotated in the pathway known to be involved in various processes of animal defense against pathogens, such as cell death/apoptosis and mitogen-activated protein kinase (MAPK). Cell death/apoptosis pathway is known to be related to the cell hypersensitivity response, blocking pathogen progression and systemic resistance[62], [63]. Among the genes involved in cell death/apoptosis, we found lysosomal aspartic protease, ATP binding cassette transmembrane transporter, caspase, beclin protein and apoptosis-inducing factor 1. Such genes may respond to the viral infection through controlling the extent of the cell death in the defense response. MAPK is another noteworthy pathway that was activated during virus infection and contributed to virus replication in animal or plant cells[64]. Among the differentially expressed genes, we found map kinase-interacting serine/threonine-protein kinase 2, heat shock protein 70, P38 mapk and polyubiquitin-C shared homology to signaling molecules of the MAPK pathway. These genes are likely to be involved in response to the TSV infection.

In summary, we employed the 454 pyrosequencing technique to investigate the transcriptome profile of *L. vanname* challenged with TSV. Comparative transcriptome analysis between TSV-infected and control groups revealed significant differences in gene expression. Although the molecular functions of some genes and their associated pathways remain largely unknown, this study provides valuable information on the antiviral mechanism in shrimp and the role of the differentially expressed genes in response to TSV infection. Furthermore, the large number of transcripts and molecular markers obtained in this study provides a strong basis for future genomic research on shrimp.

## Supporting Information

Table S1 Primers used in qRT-PCR for validation of differentially expressed genes.
(XLS)

Table S2 Length distribution of cleaned reads in the TSV infected library.
(XLS)

Table S3 Length distribution of cleaned reads in the non-infected library.
(XLS)

Table S4 GO annotation of all unigenes.
(XLS)

Table S5 KEGG annotation of all unigenes.
(XLS)

Table S6 List of differentially expressed genes with Nr, Nt, Swissprot, GO, COG, KEGG and ORF analysis.
(XLS)

Table S7 Statistics of microsatellites.
(XLS)

Table S8 PCR primers used for microsatellites.
(XLS)
